# Supplementary material for: Adolescent BCG revaccination induces a phenotypic shift in CD4+ T cell responses to Mycobacterium tuberculosis
Source: Nat Commun. 2024 Jun 18;15:5191. doi: 10.1038/s41467-024-49050-1 (PMC11189459; doi:10.1038/s41467-024-49050-1)
Supplement: Supplementary file 1 — Supplementary Information [file 41467_2024_49050_MOESM1_ESM.pdf]

O. Dintwe, et al., Adolescent BCG revaccination induces a phenotypic shift in CD4⁺ T cell responses to *Mycobacterium tuberculosis*

Supplementary Materials

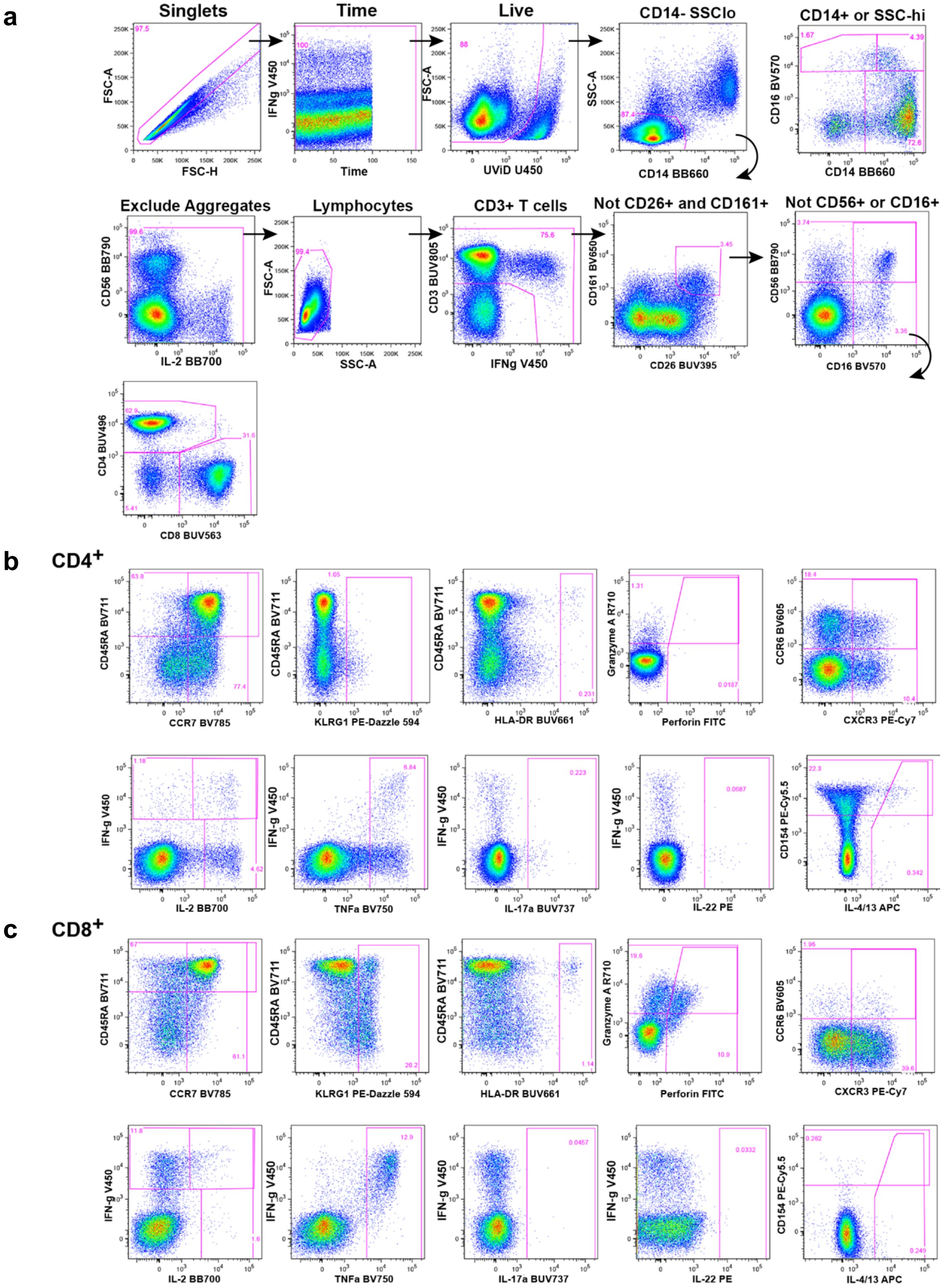

Supplementary Figure 1. 26-color ICS flow cytometry panel gating strategy. Example of the staining and gating strategy for PBMC stimulated with staphylococcal enterotoxin B (SEB). PBMC from a healthy adult were stimulated for 6 h with SEB. (A) Gating hierarchy to identify lineages. Initial singlet gating on forward scatter height vs. area, followed by a gate on time (seconds) to exclude any events early in collection if there are pressure fluctuations, live cell gating, then gating on lymphocytes. Monocytes are gated as either $CD14^+$ or high for side scatter and the upper right graph shows three monocyte subsets based on CD14 vs. CD16. Several sequential exclusion gates were included to exclude aggregates. Non-monocytes are gated as CD14-SSlo and then scatter gated on lymphocytes using FSC-A and SSC-A. Conventional $CD4^+$ and $CD8^+$ T cells are gated as $CD3^+$, then $\gamma\delta^-$, not $CD26^+$ $CD161^+$ (containing MAIT cells), and not $CD16^+$ OR $CD56^+$ (containing NKT cells). For the functional markers for (B) $CD4^+$ and (C) $CD8^+$ T cells, a gate is applied for each cytokine, and Boolean gates are created to identify cells expressing different combinations of markers.

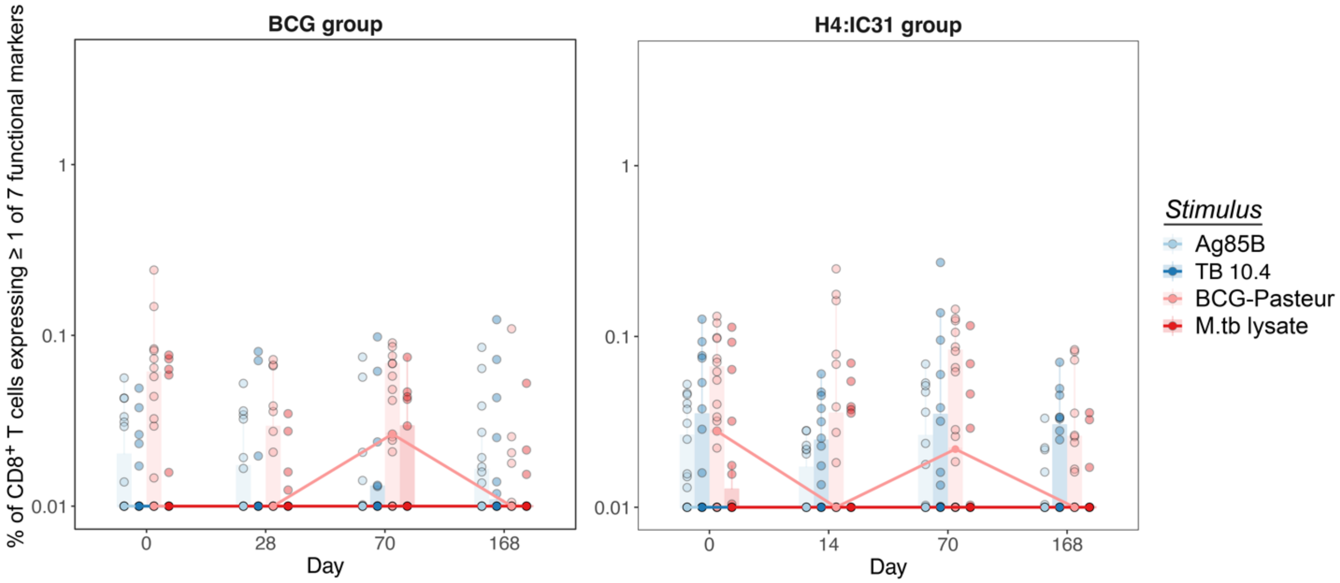

Supplementary Figure 2: No vaccine-induced change in the proportion of antigen-reactive CD8⁺ T cells. Cellular responses were evaluated by 26-color ICS. Proportion of antigen reactive CD8⁺ T cells in the BCG revaccinated (n=22 biologically independent samples), H4:IC31 vaccinated (n=24 biologically independent samples) and placebo groups (n=10 biologically independent samples) over time. Percentage of CD8⁺ T cells expressing at least 1 of the 7 functional markers (IL-2, IFN-γ, TNF-α, IL-17a, IL-4/13, IL-22 and CD154) is shown after stimulation with the Ag85B (light blue) or TB10.4 (dark blue) peptide pools, or BCG (light red), or *M.tb* lysate (dark red) in the ICS assay. Dots represent an individual participants response. Boxplots represent median responses with the first and third quartiles; whiskers extend to no further than 1.5 times the inter-quartile range. Source data are provided as a Source Data file.

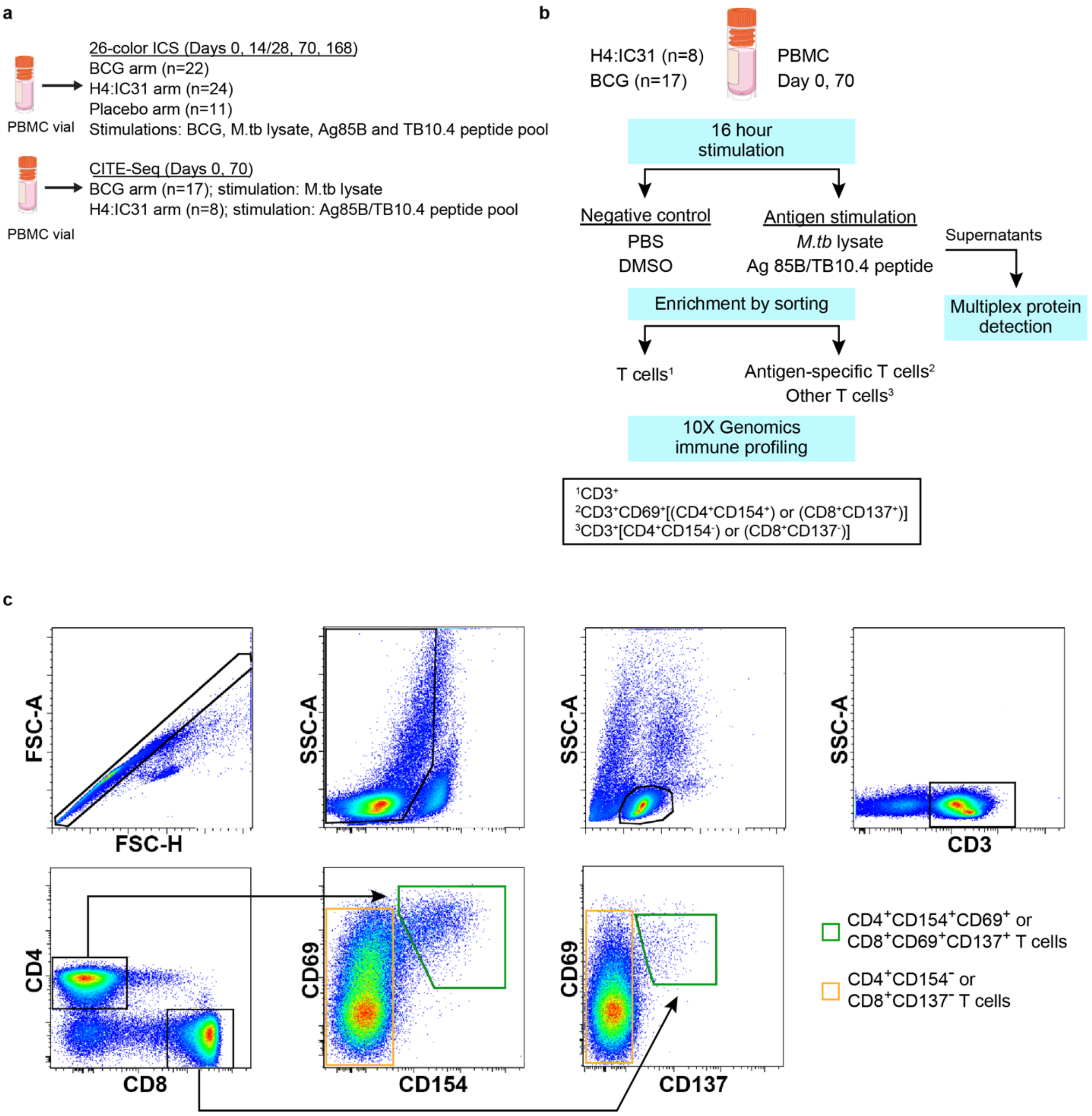

Supplementary Figure 3: Experimental design and sorting scheme. a) Cryopreserved PBMC from volunteers in HVTN 602/Aeras A-042 collected at 4 timepoints (BCG arm: days 0, 28, 70 and 168; H4:IC31 arm: days 0, 14, 70 and 168) were thawed for 26-color ICS and stimulated with 4 different antigens. For CITE-Seq, cryopreserved PBMC from days 0 and 70 were used and stimulations were limited to 2 antigens. **b**) For CITE-Seq experiments, cryopreserved PBMC were thawed and stimulated for 16 hours (BCG group: *M.tb* lysate or PBS

stimulations; H4:IC31 group: Ag85B/TB10.4 peptide pool or DMSO stimulation). Supernatants were collected for multiplex profiling of secreted cytokines and chemokines. **c**) PBMC were labeled with hashtags, oligonucleotide and/or polychromatic fluorochrome labeled antibodies/tetramer and then antigen-reactive T cells were enriched by cell sorting ($CD3^+CD69^+$ [$CD4^+CD154^+$] or [$CD8^+CD137^+$]) and $CD3^+$ (PBS/DMSO samples only) using the gating strategy shown. Sorted populations were combined as indicated in the cell hashing and pooling schema table (Supplementary Data 3) for 10X Genomics single cell profiling. A and B created with Biorender.com.

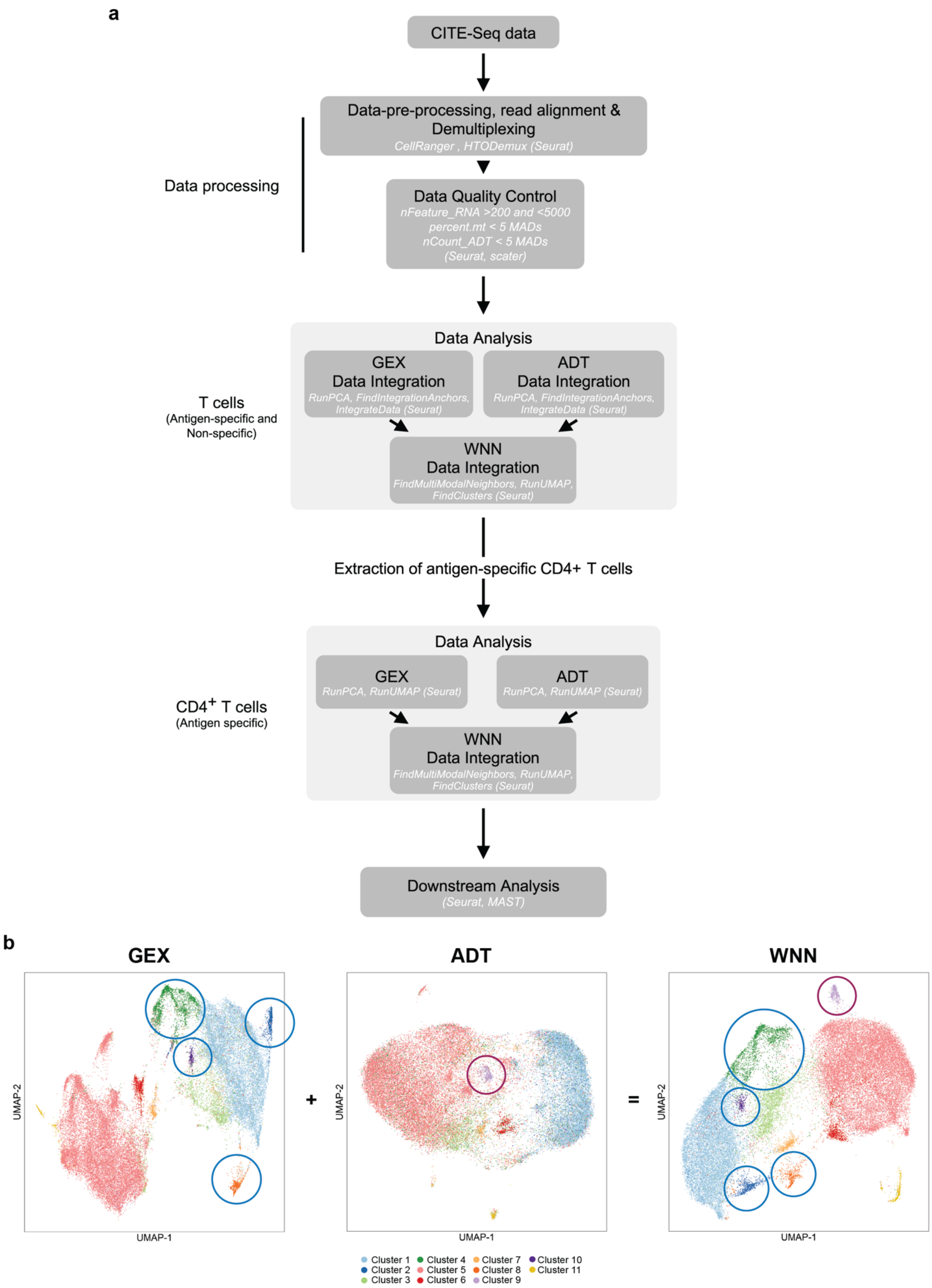
a
CITE-Seq data
Data-pre-processing, read alignment & Demultiplexing
CellRanger , HTODemux (Seurat)
Data processing
Data Quality Control
nFeature_RNA >200 and <5000
percent.mt < 5 MADs
nCount_ADT < 5 MADs
(Seurat, scater)
Data Analysis
GEX
Data Integration
RunPCA, FindIntegrationAnchors, IntegrateData (Seurat)
ADT
Data Integration
RunPCA, FindIntegrationAnchors, IntegrateData (Seurat)
T cells
(Antigen-specific and Non-specific)
WNN
Data Integration
FindMultiModalNeighbors, RunUMAP, FindClusters (Seurat)
Extraction of antigen-specific CD4+ T cells
Data Analysis
GEX
RunPCA, RunUMAP (Seurat)
ADT
RunPCA, RunUMAP (Seurat)
CD4+ T cells
(Antigen specific)
WNN
Data Integration
FindMultiModalNeighbors, RunUMAP, FindClusters (Seurat)
Downstream Analysis
(Seurat, MAST)
b
GEX
ADT
WNN
UMAP-2
UMAP-1
+
=
Cluster 1
Cluster 2
Cluster 3
Cluster 4
Cluster 5
Cluster 6
Cluster 7
Cluster 8
Cluster 9
Cluster 10
Cluster 11

Supplementary Figure 4. CITE-Seq data analysis. **a**) Flow chart highlighting CITE-Seq analysis and QC steps. Every box contains the packages, methods and criteria used for the CITE-seq analysis for data processing, alignment, counting, filtering, normalization and downstream analysis **b**) UMAPs showing clustering using gene expression alone (GEX), antibody-derived tags alone (ADT) and the combined Weighted Nearest Neighbors (WNN) approach.

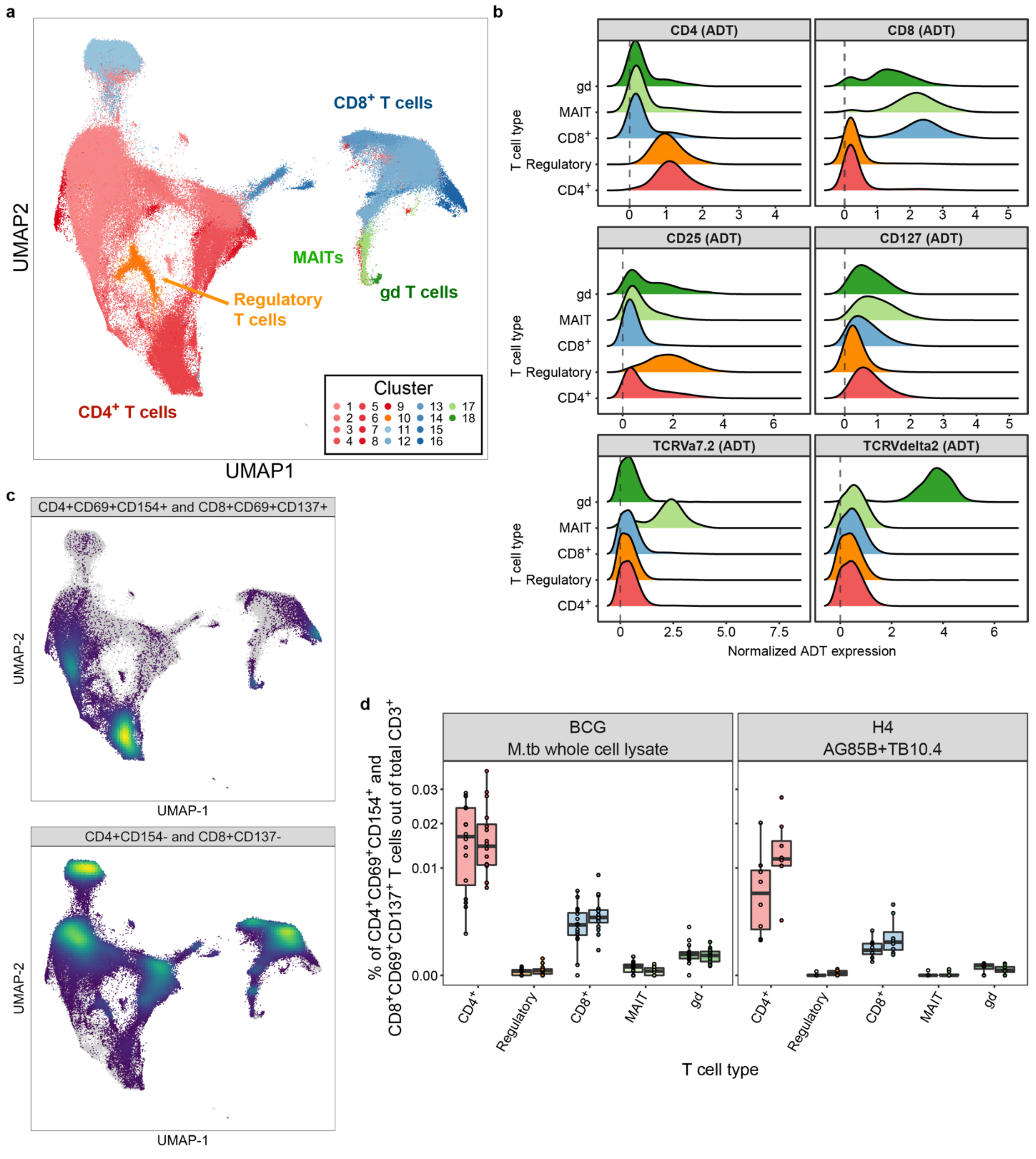

Supplementary Figure 5: Single cell proteogenomic profiling (scPGP) identifies clusters of activated T cell populations. a) Global UMAP embedding of the 241,428 CD3⁺ cells passing quality control metrics from the stimulated conditions, using the unsupervised Weighted Nearest Neighbors (WNN) clustering algorithm resulted in 18 distinct annotated clusters (CD4⁺ mega-

clusters in shades of red; $CD8^+$ mega-clusters in shades of blue; T_{reg} cells in orange, MAIT and γδ T cells in shades of green). **b**) Ridge plots of antibody-derived tags (ADT) surface markers that define broad cell lineages at the mega-cluster level to demonstrate accuracy of the cell type annotations. **c**) Density plots of antigen-reactive $CD3^+$ cells showing that Ag-reactive sorted cells ($CD4^+CD69^+CD154^+$ or $CD8^+CD69^+CD137^+$) fall into distinct clusters from other $CD3^+$ cells. **d**) Boxplots showing the proportion of antigen-reactive T cells on a per participant/visit level at the mega-cluster level (unfilled circles, Day 0; filled circles, Day 70). Boxplots indicate the median response and the first and third quartiles; whiskers extend to no further than 1.5 times the inter-quartile range.

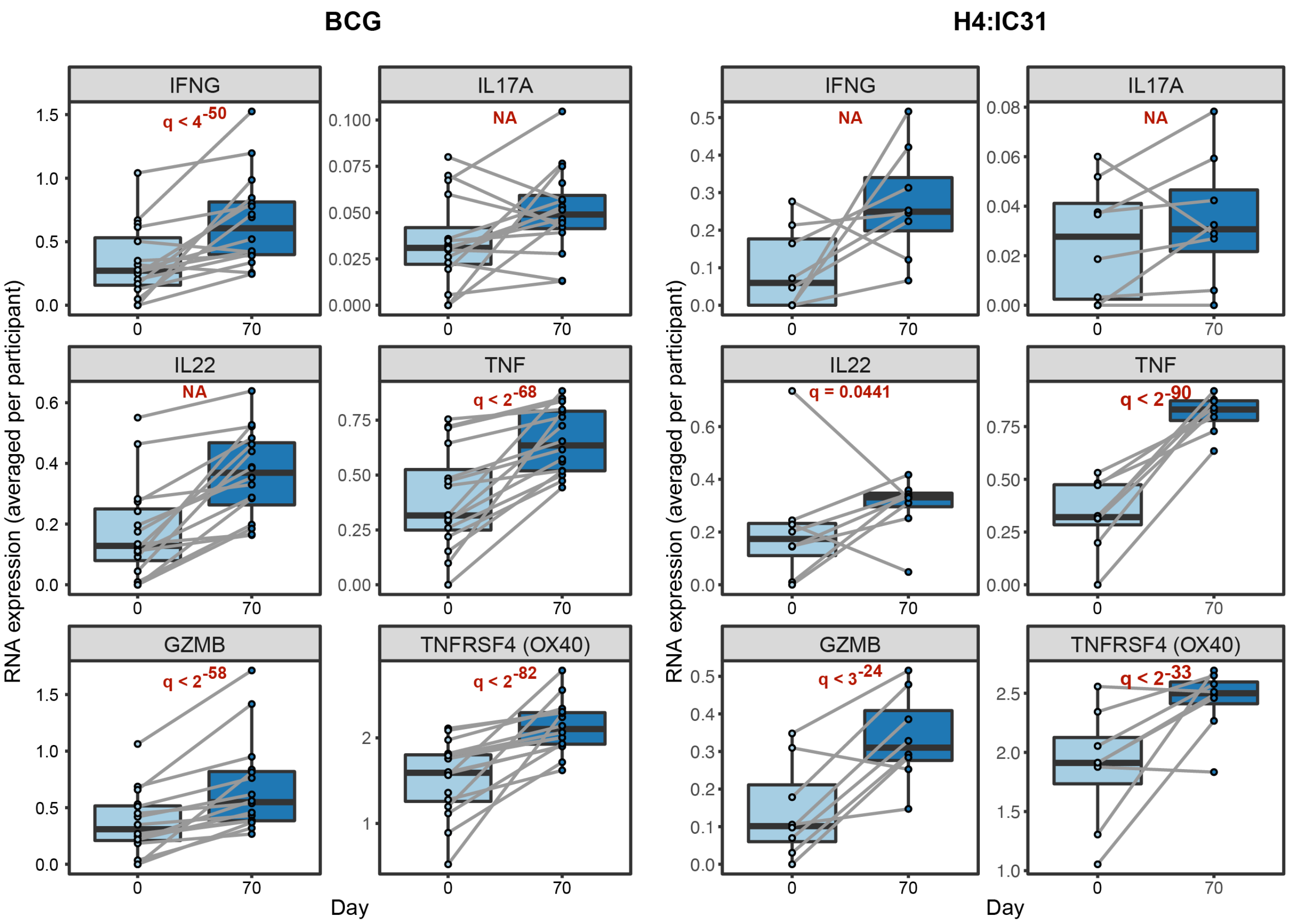

Supplementary Figure 6: BCG re-vaccination and H4:IC31 vaccination increase effector gene expression. Boxplots showing differentially expressed genes that were upregulated 70 days post vaccination (Day 70, dark blue) compared to baseline (Day 0, light blue) in both BCG and H4:IC31 vaccine groups. Each point depicts the response in a single participant. There are no q-values for IFNG & IL-17A in H4:IC31, nor for IL-17A & IL-22 in BCG, because these genes were not expressed in more than 10% of the samples, a requirement to perform the statistical test. Boxplots indicate the median response and the first and third quartiles; whiskers extend to no further than 1.5 times the inter-quartile range.

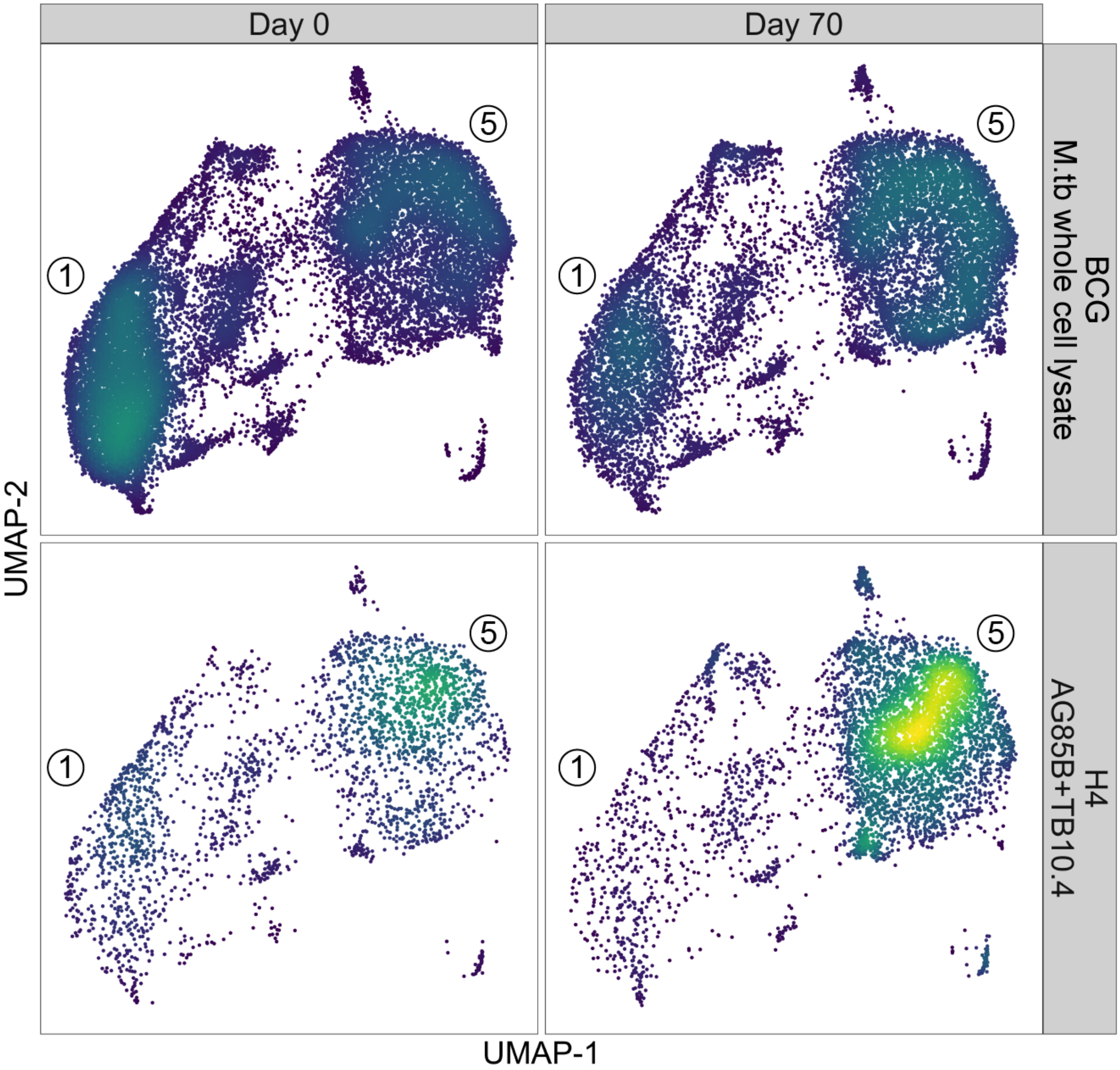

Supplementary Figure 7: TB vaccination induces distinct clusters of CD4$^+$ T cells. Density plot of showing all CD4$^+$CD69$^+$CD154$^+$ measured by CITE-Seq, by vaccine arm and timepoint. 2D kernel density estimation was used to estimate the point density.

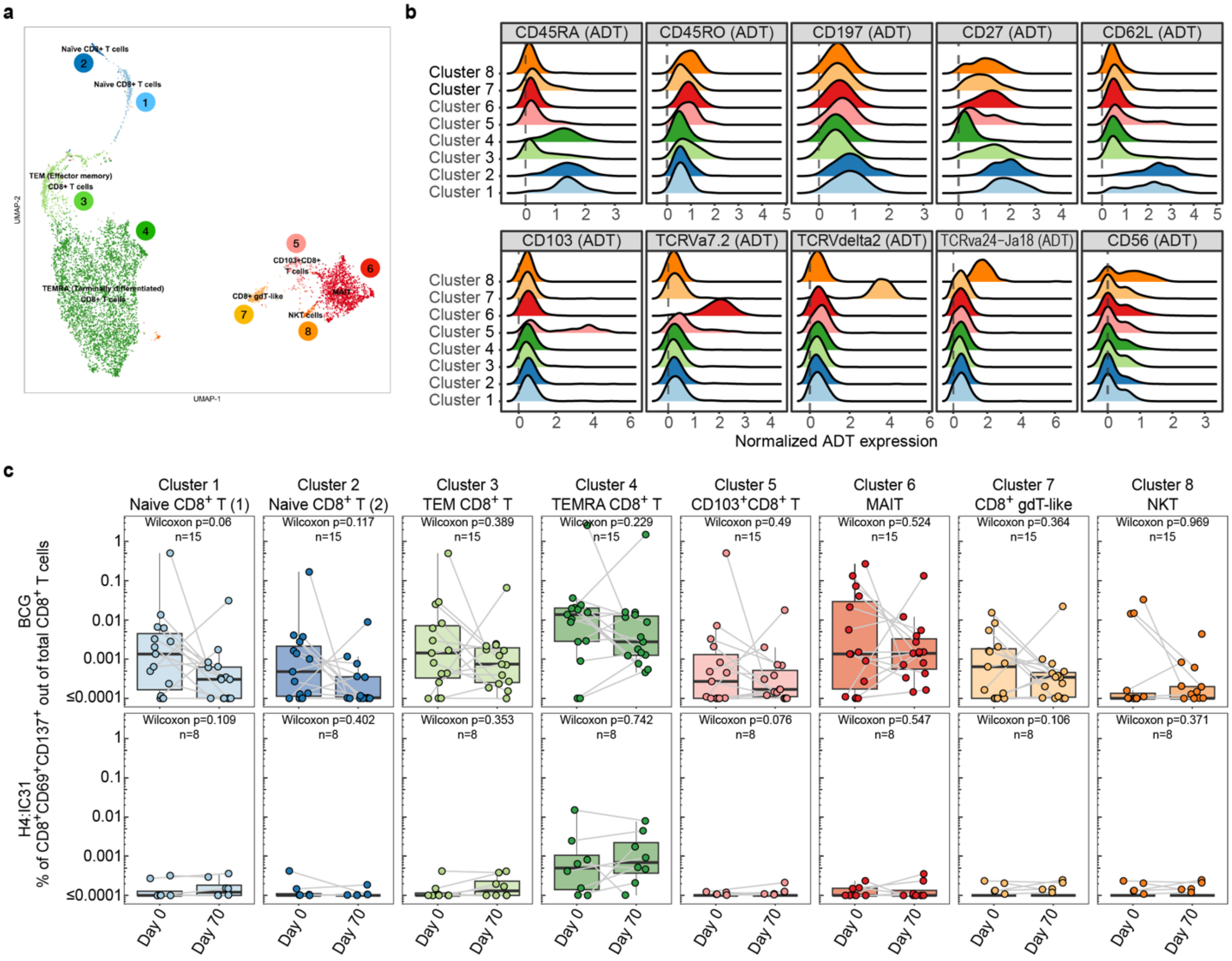

Supplementary Figure 8: BCG vaccination induces distinct clusters of CD8⁺ T cells. a) Unsupervised WNN clustering and UMAP projection of ~8000 CD8⁺ (CD69⁺CD137⁺) activated T cells measured by CITE-Seq reveals 8 distinct clusters. **b**) Ridge plot of the normalized antibody-derived tagged (ADT) surface marker expression on antigen-reactive CD8⁺ T cells by cluster. **c**) Proportion CD8⁺CD69⁺CD137⁺ T cells of total CD8⁺ T cells present in each cluster, measured by CITE-Seq, after vaccination in either the BCG (n = 15) or the H4:IC31 (n = 8) vaccine groups. *p<0.05, **p<0.01, two-sided Wilcoxon Signed Rank test. Each point depicts the response in a single participant. Boxplots indicate the median response and the first and third quartiles; whiskers extend to no further than 1.5 times the inter-quartile range.

a

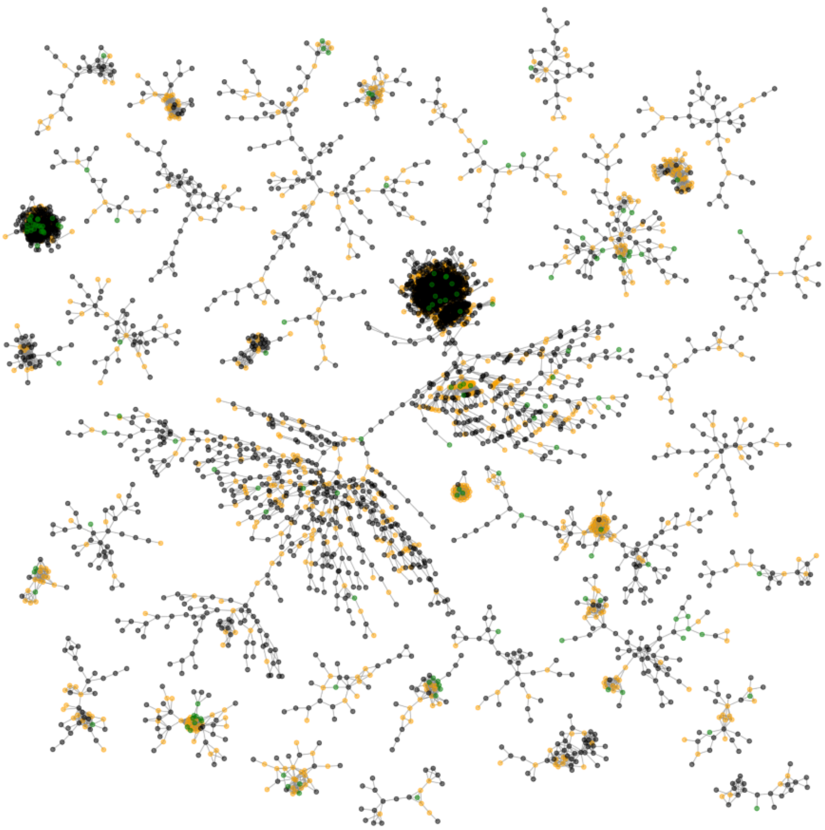

b

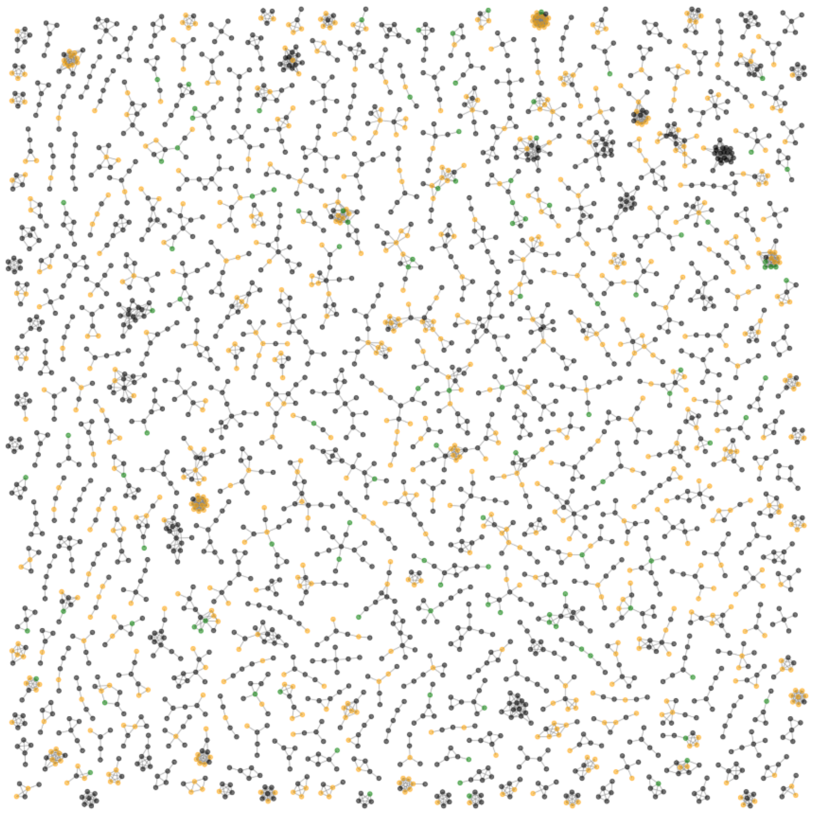

Supplementary Figure 9: TCR repertoire analysis. a) Network of public TCRs with 5-20 neighbors from the current dataset (black nodes), and two published datasets (green nodes,

Huang et al; yellow nodes, Musvosvi, et al.). Network edges shown are those formed between TCRs in different participants. **b**) Network of public TCRs with >20 neighbors. The largest clusters likely represent donor-unrestricted T cells, whereas the others may represent TCRs reactive to dominant *M.tb* epitopes.

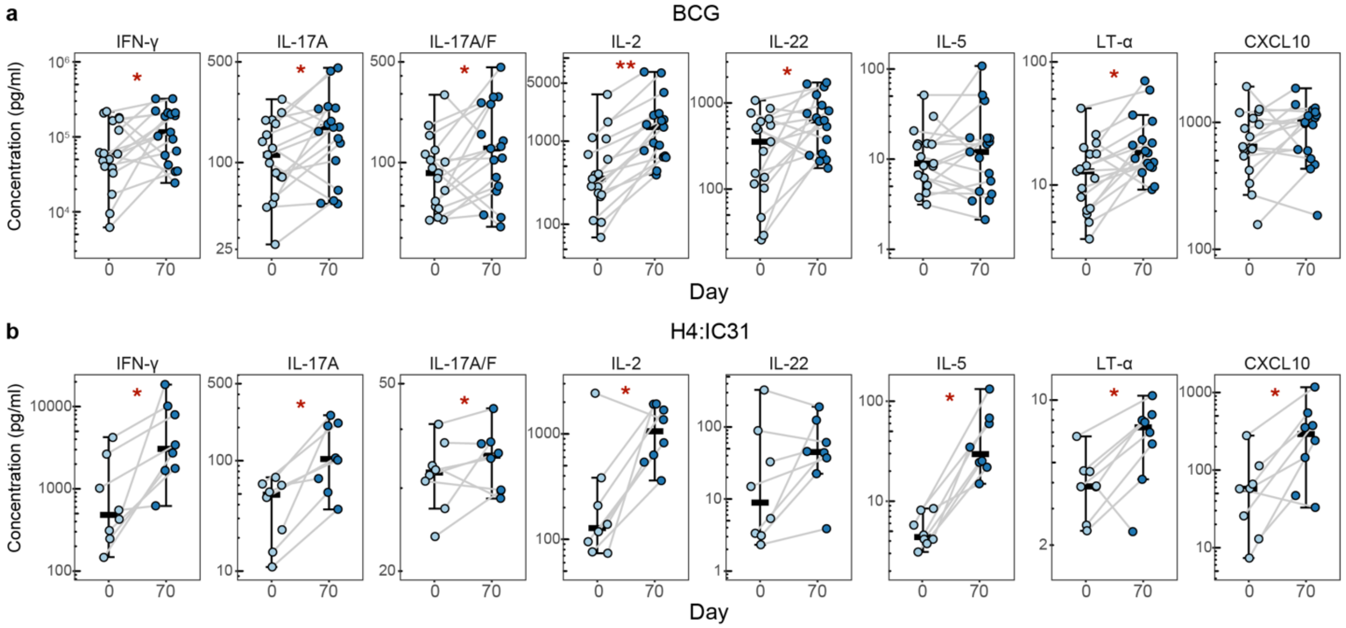

Supplementary Figure 10: BCG re-vaccination and H4:IC31 vaccination increase secretion of Th1 and Th17 cytokines and chemokines from stimulated PBMC. Secreted cytokine/chemokine concentrations after stimulation of PBMC from volunteers in the BCG revaccination group (**a**) or H4:IC31 vaccination group (**b**) for 16 hours for the CITE-Seq experiments. Each point depicts the response in a single participant. Boxplots indicate the median response and the first and third quartiles; whiskers extend to no further than 1.5 times the inter-quartile range. * FDR <0.2, **FDR <0.05. Multiplicity adjustments were made over the number of analytes within each combination of group and stimulation.
