# Supplementary material for: Adolescent BCG revaccination induces a phenotypic shift in CD4+ T cell responses to Mycobacterium tuberculosis
Source: Nat Commun. 2024 Jun 18;15:5191. doi: 10.1038/s41467-024-49050-1 (PMC11189459; doi:10.1038/s41467-024-49050-1)
Supplement: Supplementary file 3 — Description of Additional Supplementary Files [file 41467_2024_49050_MOESM3_ESM.pdf]

Description of Additional Supplementary Files

Supplementary Data 1: Comparison of background subtracted median frequencies of responding CD4+ T-cells across timepoints to baseline (pre-vaccination). The Wilcoxon signed-rank test was used for comparisons and only significant comparisons are shown, FDR q-value < 0.05.

Supplementary Data 2: Comparison of background subtracted median frequencies of responding CD4+ T-cells in the vaccination groups following stimulation. The two sided Wilcoxon signed-rank test was used for comparisons and only significant comparisons are shown, FDR q-value < 0.05.

Supplementary Data 3: Comparisons of post vaccination timepoints to baseline for each group using Phenograph in Figure 2B and C. The two-sided Wilcoxon signed-rank test was used for comparisons and only significant comparisons are shown, FDR q-value < 0.05.

Supplementary Data 4: Bulk gene expression changes in CD4+ T cells (Day 0 vs 70) in H4:IC31 and BCG groups for the top 20 genes. Differential expression analysis was performed using the R package MAST (Finak et al., 2015). Pr(>Chisq) corresponds to likelihood ratio test p-value; coef corresponds to the log(2) fold changes from hurdle model components.

Supplementary Data 5: ADT markers for each CD4+ T cluster. To identify ADT cluster markers, the FindAllMarkers R function was used with test.use = LR, latent.vars = c("batch", "ptid", "arm", "stimulation", "visit"), and max.cells.per.ident = 5000. avglog2FC: log2 fold-change of the average expression between the two groups. Positive values indicate that the protein is more highly expressed in the first group. FWER: Family wise error rate, adjusted p-value, based on Bonferroni correction using all proteins in the dataset. pct.1 & pct.2: Proportion of cells in which the ADT is detected in the first & and second groups, where the two groups are the cluster versus all other clusters combined.

Supplementary Data 6: Gene markers for each CD4+ T cluster compared to all other clusters. To identify gene cluster markers, the FindAllMarkers R function was used with test.use =

MAST, latent.vars = c("nCount_RNA", "batch", "ptid", "arm", "stimulation", "visit"), and max.cells.per.ident = 5000. avglog2FC: log2 fold-change of the average expression between the two groups. Positive values indicate that the gene is more highly expressed in the first group. FWER: Family wise error rate, Adjusted p-value, based on Bonferroni correction using all genes in the dataset. pct.1 & pct.2: Proportion of cells in which the gene is detected in the first & and second groups, where the two groups are the cluster versus all other clusters combined.

Supplementary Data 7: Differentially expressed ADTs between Cluster 1 and 2. To identify differentially expressed ADTs between cluster 1 and 2, the FindMarkers R function was used with ident.1 = "Seurat 1", ident.1 = "Seurat 2", test.use = LR and latent.vars = c("batch", "ptid", "arm", "stimulation", "visit"). avglog2FC: log2 fold-change of the average expression between the two groups. Positive values indicate that the protein is more highly expressed in the first group. FWER: Family wise error rate, adjusted p-value, based on Bonferroni correction using all proteins in the dataset. pct.1 & pct.2: Proportion of cells in which the ADT is detected in the first & and second groups, where the two groups are the Cluster 1 versus Cluster 2.

Supplementary Data 8: Differentially expressed genes between Cluster 1 and 2. To identify differentially expressed genes between cluster 1 and 2, the FindMarkers R function was used with ident.1 = "Seurat 1", ident.1 = "Seurat 2", test.use = MAST and latent.vars = c("nCount_RNA", "batch", "ptid", "arm", "stimulation", "visit"). avglog2FC: log2 fold-change of the average expression between the two groups. Positive values indicate that the gene is more highly expressed in the first group. FWER: Family wise error rate, adjusted p-value, based on Bonferroni correction using all genes in the dataset. pct.1 & pct.2: Proportion of cells in which the gene is detected in the first & and second groups, where the two groups are the Cluster 1 versus Cluster 2.

Supplementary Data 9: Gene markers for each EM CD4+ T cluster. To identify gene cluster markers, the FindAllMarkers R function was used with test.use = MAST, latent.vars = c("nCount_RNA", "batch", "ptid", "arm", "stimulation", "visit"), and max.cells.per.ident = 5000. avglog2FC: log2 fold-change of the average expression between the two groups. Positive values indicate that the gene is more highly expressed in the first group. FWER: Family

wise error rate, adjusted p-value, based on Bonferroni correction using all genes in the dataset. pct.1 & pct.2: Proportion of cells in which the gene is detected in the first & and second groups, where the two groups are the cluster versus all other clusters combined.

Supplementary Data 10: ADT markers for each EM CD4+ T cluster. To identify ADT cluster markers, the FindAllMarkers R function was used with test.use = LR, latent.vars = c("batch", "ptid", "arm", "stimulation", "visit"), and max.cells.per.ident = 5000. avglog2FC: log2 fold-change of the average expression between the two groups. Positive values indicate that the protein is more highly expressed in the first group. FWER: Family wise error rate, adjusted p-value, based on Bonferroni correction using all proteins in the dataset. pct.1 & pct.2: Proportion of cells in which the ADT is detected in the first & and second groups, where the two groups are the cluster versus all other clusters combined.

Supplementary Data 11: ADT markers for each CD8+ T cluster. To identify ADT cluster markers, the FindAllMarkers R function was used with test.use = LR, latent.vars = c("batch", "ptid", "arm", "stimulation", "visit"). avglog2FC: log2 fold-change of the average expression between the two groups. Positive values indicate that the protein is more highly expressed in the first group. FWER: Family wise error rate, adjusted p-value, based on Bonferroni correction using all proteins in the dataset. pct.1 & pct.2: Proportion of cells in which the ADT is detected in the first & and second groups, where the two groups are the cluster versus all other clusters combined

Supplementary Data 12: Gene markers for each CD8+ T cluster compared to all other clusters. To identify gene cluster markers, the FindAllMarkers R function was used with test.use = MAST, latent.vars = c("nCount_RNA", "batch", "ptid", "arm", "stimulation", "visit"). avglog2FC: log2 fold-change of the average expression between the two groups. Positive values indicate that the gene is more highly expressed in the first group. FWER: Family wise error rate, adjusted p-value, based on Bonferroni correction using all genes in the dataset. pct.1 & pct.2: Proportion of cells in which the gene is detected in the first & and second groups, where the two groups are the cluster versus all other clusters combined.

Supplementary Data 13: Cytokine and chemokine concentrations in supernatants from stimulated PBMC from day 0 and 70.

Supplementary Data 14: Demographics of participants from whom samples were assayed in this study.

Supplementary Data 15: Antibody panel used to perform the PBMC intracellular cytokine staining assay.

Supplementary Data 16: Oligo labelled antibodies and tetramer used for the CITE-seq panel.

Supplementary Data 17: Fluorochrome-labelled antibodies used for enrichment of activated T cells and CITE-seq.

Supplementary Data 18: Cell hashing schema.

Supplementary Table 19: Kits used for the T cell receptor repertoire sequencing.
