# Supplementary material for: Adolescent BCG revaccination induces a phenotypic shift in CD4+ T cell responses to Mycobacterium tuberculosis
Source: Nat Commun. 2024 Jun 18;15:5191. doi: 10.1038/s41467-024-49050-1 (PMC11189459; doi:10.1038/s41467-024-49050-1)
Supplement: Supplementary file 5 — Reporting Summary [file 41467_2024_49050_MOESM5_ESM.pdf]

nature portfolio

Corresponding author(s): Margaret Juliana McElrath

Last updated by author(s): Jan 12, 2024

Reporting Summary

Nature Portfolio wishes to improve the reproducibility of the work that we publish. This form provides structure for consistency and transparency in reporting. For further information on Nature Portfolio policies, see our Editorial Policies and the Editorial Policy Checklist.

Statistics

For all statistical analyses, confirm that the following items are present in the figure legend, table legend, main text, or Methods section.

n/a	Confirmed	
☐	☒	The exact sample size (n) for each experimental group/condition, given as a discrete number and unit of measurement
☒	☐	A statement on whether measurements were taken from distinct samples or whether the same sample was measured repeatedly
☐	☒	The statistical test(s) used AND whether they are one- or two-sided<br>*Only common tests should be described solely by name; describe more complex techniques in the Methods section.*
☒	☐	A description of all covariates tested
☐	☒	A description of any assumptions or corrections, such as tests of normality and adjustment for multiple comparisons
☐	☒	A full description of the statistical parameters including central tendency (e.g. means) or other basic estimates (e.g. regression coefficient) AND variation (e.g. standard deviation) or associated estimates of uncertainty (e.g. confidence intervals)
☐	☒	For null hypothesis testing, the test statistic (e.g. F, t, r) with confidence intervals, effect sizes, degrees of freedom and P value noted<br>*Give P values as exact values whenever suitable.*
☒	☐	For Bayesian analysis, information on the choice of priors and Markov chain Monte Carlo settings
☒	☐	For hierarchical and complex designs, identification of the appropriate level for tests and full reporting of outcomes
☐	☒	Estimates of effect sizes (e.g. Cohen's d, Pearson's r), indicating how they were calculated

Our web collection on statistics for biologists contains articles on many of the points above.

Software and code

Policy information about availability of computer code

Data collection	BD FACS DIVA v8.0.1<br>MESO QuickPlex SQ 120 platform software v4.0.12
Data analysis	Flowjo, v9.9.6<br>R package uwot, version 0.1.16<br>PhenoGraph algorithm (Levine et al., Cell 2015), version 0.99.1<br>Cell Ranger Single-Cell Software (version 3.1.0)<br>Seurat package (version 4.0.1), HTODEMUX<br>R package MAST ,version 1.26.0<br>nCal R software package (Fong et al., Bioinformatics 2013)<br>Code developed for this work can be found here: https://github.com/ValentinVoillet/BCG_revax (DOI: 10.5281/zenodo.10362451)

For manuscripts utilizing custom algorithms or software that are central to the research but not yet described in published literature, software must be made available to editors and reviewers. We strongly encourage code deposition in a community repository (e.g. GitHub). See the Nature Portfolio guidelines for submitting code & software for further information.

Data

Policy information about availability of data

All manuscripts must include a data availability statement. This statement should provide the following information, where applicable:

- Accession codes, unique identifiers, or web links for publicly available datasets
- A description of any restrictions on data availability
- For clinical datasets or third party data, please ensure that the statement adheres to our policy

All the data supporting this study have been deposited in an archive on figshare and are publicly available using this hyper link: https://figshare.com/articles/dataset/HVTN_602_data_repo_Adolescent_BCG_revaccination_induces_a_phenotypic_shift_in_CD4_T_cell_responses_to_Mycobacterium_tuberculosis/24150492 . The raw transcriptomic data are protected and are not available due to data privacy laws; the processed transcriptomics data are provided as aggregated gene counts per cell in the archive above.
In addition the raw FCS files from the flow cytomerty based experiments can be found on the FlowRepository, ID: FR-FCM-Z752.

Research involving human participants, their data, or biological material

Policy information about studies with human participants or human data. See also policy information about sex, gender (identity/presentation), and sexual orientation and race, ethnicity and racism.

Reporting on sex and gender	Data were previously reported in the primary clinical trial publication which has been referenced in this manuscript (Bekker et al.,). Data for a subset of those participants for who we used samples in this study is in Supplemental Data 14.
Reporting on race, ethnicity, or other socially relevant groupings	Data were previously reported in the primary clinical trial publication which has been referenced in this manuscript (Bekker et al.,). In the parent study, 98.8% of the trial participants were black. For the participants whom we used samples from for this study, 100% were black.
Population characteristics	Healthy, HIV and QuantiFERON-negative adolescents aged 12-17 who had received BCG vaccination (SSI, 2-8×10^5 cfu) at birth were enrolled in the trial. Further characteristics are in the primary clinical trial publication which has been referenced in this manuscript (Bekker et al., EClinMed 2020).
Recruitment	Data were previously reported in the primary clinical trial publication which has been referenced in this manuscript (Bekker et al., EClinMed 2020).
Ethics oversight	The study was approved by the University of Cape Town Ethics Committee. The HVTN 602 trial is registered with the U.S. National Institutes of Health Clinical Trials Registry (ClinicalTrials.gov identifier NCT02378207).

Note that full information on the approval of the study protocol must also be provided in the manuscript.

Field-specific reporting

Please select the one below that is the best fit for your research. If you are not sure, read the appropriate sections before making your selection.

☒ Life sciences ☐ Behavioural & social sciences ☐ Ecological, evolutionary & environmental sciences

For a reference copy of the document with all sections, see nature.com/documents/nr-reporting-summary-flat.pdf

Life sciences study design

All studies must disclose on these points even when the disclosure is negative.

Sample size	Overall trial samples size data were previously reported in the primary clinical trial publication which has been referenced in this manuscript (Bekker et al. EClinMed, 2020). Briefly, 24 participants were recruited to receive H4:IC31, another 24 were vaccinated with BCG and 12 participants received the placebo. For this work, sample sizes for each experiment were determined by the availability of PBMC to perform the work
Data exclusions	Data exclusion for each of the assays is described in the assay relevant methods section. For the CITE-Seq analysis, data were excluded as per the QC steps described in Supplementary Figure 4. Quality control for the data was performed as follows: following sequence alignment and demultiplexing, cells with high (>5000) feature counts (as proxy for exclusion of droplets containing more than one cell) or very low (less than 200) (as proxy for exclusion of empty droplets) were excluded 65. Cells with high mitochondrial proportions (as proxy for cell damage) were also filtered out. For each combination of batch and pool, a threshold of 5 Median Absolute Deviation (MAD) for percent of mitochondrial genes was used. Cells with ADT counts were retained; whereas cells with unusual low numbers of detected ADTs, defined here as half of the median across all cells for each combination of batch and pool, were removed (57 cells were filtered out at this step). As above, we also used a generous cutoff (5 MADs) to exclude cells with large ADT counts (as proxy for "sticky" cells).
Replication	No replication was done. Replicates are not typically run for in the ICS assay performed for clinical trials due to the robustness of our ICS assay and due to cost associated with run replicates.

Randomization	Randomisation for the clinical trial has been described in the primary clinical trial publication which has been referenced in this manuscript (Bekker et al. EClinMed, 2020). Participants were randomized to 1 of 3 treatment groups or the control group in a 2:2:2:1 ratio. The randomization sequence was computer-generated and provided to the clinical research site through an interactive web response system (IWRS) developed and managed by Almac (https://www.almacgroup.com/). The randomization schedule was prepared by a statistician who was not involved in the analysis of the study in order to maintain blinding of the study team. The randomization was done in blocks to ensure balance across arms over time.
Blinding	Blinding was performed for the clinical trial as has been described in the primary clinical trial publication which has been referenced in this manuscript (Bekker et al. EClinMed, 2020). The CRS pharmacist with primary responsibility for dispensing study products was charged with maintaining security of the treatment assignments. This was a partially blinded trial, as H4:IC31, H56:IC31 and placebo were administered in a blinded fashion; given that BCG is administered by a different route and is associated with well-characterized vaccination site reactions, BCG was administered in an unblinded fashion by a staff member not otherwise involved in the study. The day of enrolment for each participant was study day 0, and participants who discontinued from the trial were not replaced. In addition, investigators performing assays for this manuscript were blinded until data analysis was performed when they were then unblinded to be able to assign participants to the different vaccine arms.

Reporting for specific materials, systems and methods

We require information from authors about some types of materials, experimental systems and methods used in many studies. Here, indicate whether each material, system or method listed is relevant to your study. If you are not sure if a list item applies to your research, read the appropriate section before selecting a response.

Materials & experimental systems

n/a	Involved in the study	
☐	☒	Antibodies
☒	☐	Eukaryotic cell lines
☒	☐	Palaeontology and archaeology
☒	☐	Animals and other organisms
☐	☒	Clinical data
☒	☐	Dual use research of concern
☒	☐	Plants

Methods

n/a	Involved in the study	
☒	☐	ChIP-seq
☐	☒	Flow cytometry
☒	☐	MRI-based neuroimaging

Antibodies

Antibodies used	Perforin-FITC (clone B-D48, Biolegend cat no. 353310), CD14 BB660 (clone: MΦP9, BD Horizon catalog no: 624295), IL-2 BB700 (clone: MQ1-17H12, BD Horizon catalog no: 566405), CD56 BB790 (clone: NCAM16.2, BD Custom catalog no: 624296), IL-22 PE (clone: 22URTI, ebioscience catalog no: 12-7229-42), KLRG1 PE-Dazzle 594 (clone: SA231A2, Biolegend catalog no: 367710), CD154 PE-Cy5 (clone: TRAP1, BD Pharm catalog no. 555701), CXCR3 (CD183) PE-Cy7 (clone: 1C6/CXCR3, BD Pharm catalog no: 560831), IL-4 APC (clone; MP4-25D2, Biolegend catalog no. 500812), IL-13 APC (clone; JES10-5A2, BD Pharm catalog no. 561152), GranzymeA-Alx700 (clone: CB9, BD Pharm catalog no: 624358), TCRv7.2 APC-Cy7 (clone: 3C10, Biolegend catalog no: 351714), CD3 BUV805 (clone SK7, BD Horizon catalog no: 565515), CD45RA BV711 (clone: HI100, BD Horizon catalog no. 563733), CD8 BUV563 (clone: RPA-T8, BD Horizon catalog no. 564804), HLA-DR BUV661 (clone: G46-6, BD Horizon catalog no. 565073), IL-17a BUV737 (clone: N49-653, BD Horizon catalog no. 624286), CD4 BUV496 (clone: SK3, BD Custom catalog no. 705772), IFN- V450 (clone: B27, BD Pharm catalog no. 560371), TCRBV510 (clone: B1, Biolegend catalog no. 331220), CD16 BV570 (clone: 3G8, Biolegend catalog no. 302036), CCR6 (CD196) BV605 (clone: 11-A9 BD Horizon catalog no. 562724), CD161 BV650 (clone: DX12, BD Horizon catalog no. 563864), CD26 BUV395 (clone: M-A261 BD Optibuild catalog no. 744454), TNF-a BV750 (clone: Mab11 BD Horizon 566359), CCR7 (CD197) BV785 (clone: G043H7 Biolegend catalog no. 353230).<br><br>Antibodies are listed in Supplementary Data 14.
Validation	Antibodies used are Research Use Only (RUO) that are available commercially. Antibodies were titrated and FMO experiments performed to optimize panel performance. Panel was then tested prior to use in the study samples.

Clinical data

Policy information about clinical studies

All manuscripts should comply with the ICMJE guidelines for publication of clinical research and a completed CONSORT checklist must be included with all submissions.

Clinical trial registration	ClinicalTrials.gov Identifier: NCT02378207
Study protocol	https://clinicaltrials.gov/ct2/show/NCT02378207
Data collection	Described in the primary clinical trial publication which has been referenced in this manuscript (Bekker et al., EClinMed., 2020). The HVTN 602/Aeras A-042 study was conducted at the Emavundleni Clinical Research Site (CRS) in Crossroads, Cape Town, South Africa. 84 study participants were randomized into the study between July 2015 and March 2016.
Outcomes	Outcomes described in this paper are from exploratory immunology assessments from a subset of the HVTN 602 clinical trial and had no pre-defined outcomes.

Flow Cytometry

Plots

Confirm that:

- [x] The axis labels state the marker and fluorochrome used (e.g. CD4-FITC).
- [x] The axis scales are clearly visible. Include numbers along axes only for bottom left plot of group (a 'group' is an analysis of identical markers).
- [x] All plots are contour plots with outliers or pseudocolor plots.
- [x] A numerical value for number of cells or percentage (with statistics) is provided.

Methodology

Sample preparation	Briefly, cryopreserved PBMC were thawed, incubated overnight and stimulated on day 2 for six hours at 37°C with either peptide pools (peptides of 15 amino acids overlapping in sequence by 11 amino acids) for the vaccine-matched proteins (Ag85B and TB10.4, final concentration of 1 µg/ml in DMSO, Bio-Synthesis Inc.), BCG (Pasteur strain from glycerol stocks at a final MOI of 9 [provided by Aeras]), gamma-irradiated M.tb H37Rv whole cell lysate (BEI resources, cat NR-14822, final concentration of 30 µg/ml), dimethyl sulfoxide (DMSO, 0.5%, Sigma Aldrich; negative control) or staphylococcal enterotoxin B (SEB, 0.25 µg/ml; Sigma Aldrich; positive control) in the presence of costimulatory antibodies CD28 and CD49d (1 µg/ml, BD Biosciences) and brefeldin A (BFA, 10 µg/ml, Sigma Aldrich). Cells were incubated with EDTA (2 mM, Life Technologies) overnight at 4°C, then stained with a 26-color antibody staining panel
Instrument	BD FACSymphony A5 flow cytometer (BD Biosciences)
Software	FlowJo version 9.9.6
Cell population abundance	All sorted cell populations had purities >90%
Gating strategy	Example of the staining and gating strategy for PBMC stimulated with staphylococcal enterotoxin B (SEB). PBMC from a healthy adult were stimulated for 6 h with SEB. (A) Gating hierarchy to identify lineages. Initial singlet gating on forward scatter height vs. area, followed by a gate on time (seconds) to exclude any events early in collection if there are pressure fluctuations, live cell gating, then gating on lymphocytes. Monocytes are gated as either CD14+ or high for side scatter and the upper right graph shows three monocyte subsets based on CD14 vs. CD16. Several sequential exclusion gates were included to exclude aggregates. Non-monocytes are gated as CD14-SSlo and then scatter gated on lymphocytes using FSC-A and SSC-A.<br><br>The gating scheme avoids any overlapping subsets as shown in supplemental figure 4. Thus, conventional CD4+ and CD8+ T cells are gated as CD3+, then γδ-, not CD26 + CD161+ (containing MAIT cells), and not CD16+ OR CD56+ (containing NKT cells). For the functional markers for CD4+ and CD8+ T cells, a gate is applied for each cytokine, and Boolean gates are created to identify cells expressing different combinations of markers. The single function gates are sometimes chosen vs. a parameter that displays some FMO spreading to allow for angled gates. Most gates are copied, applied to all lineages, and then cloned so that any changes to the gate on one lineage changes that gate for all lineages. However, the IL-22 gate was uniquely lower for CD4 T cells (compared to other lineages) since the CD8 reagent caused some spreading into IL-22 and thus requiring a higher gate for all other lineages that express CD8.<br><br>Additional functional and non-functional markers for CD4+ and CD8+ T cells. Perforin and granzyme A are constitutive but can be examined as co-expression with another functional marker.

- [x] Tick this box to confirm that a figure exemplifying the gating strategy is provided in the Supplementary Information.
